# Disseminated *Echinococcus multilocularis* Infection without Liver Involvement in Child, Canada, 2018

**DOI:** 10.3201/eid2608.191644

**Published:** 2020-08

**Authors:** Joanna Joyce, Xiao-Ou He, Katya Rozovsky, Camelia Stefanovici, Sergio Fanella

**Affiliations:** Memorial University, St. John’s, Newfoundland, Canada (J. Joyce);; University of Manitoba, Winnipeg, Manitoba, Canada (X.-O. He, K. Rozovsky, C. Stefanovici, S. Fanella)

**Keywords:** Echinococcus multilocularis, alveolar, hydatid, liver, children, zoonoses, parasites, Canada

## Abstract

An immunocompetent child in Canada received a diagnosis of disseminated alveolar *Echinococcus* *multilocularis* infection. The case lacked typical features of liver involvement and was possibly related to a rare congenital portosystemic shunt. We summarize the rapidly evolving epidemiology of *E. multilocularis* parasites in Canada.

Alveolar echinococcosis (AE) is a zoonosis caused by the metacestode of *Echinococcus multilocularis* parasites*.* The definitive hosts include foxes and canines; humans become infected through accidental ingestion of ova dispersed in the environment from feces. AE affects mainly adults, by the formation of irregular lesions within various organs, primarily the liver (*1*,*2*). In Canada, the incidence of human cases of *E. multilocularis* is low (*3*). We describe a case of disseminated AE without hepatic involvement in a child from Manitoba with a congenital portosystemic shunt, in the context of emerging epidemiology of AE in Canada.

## The Study

A 12-year-old boy from a remote community who had a history of latent tuberculosis treated 10 years previously was brought for care of 2 months of painless gross hematuria. His only other exposure history was occasionally playing with local stray dogs. He denied other symptoms. Results of his physical examination were unremarkable aside from a BMI of 35.4 kg/m^2^.

**Figure 1:**
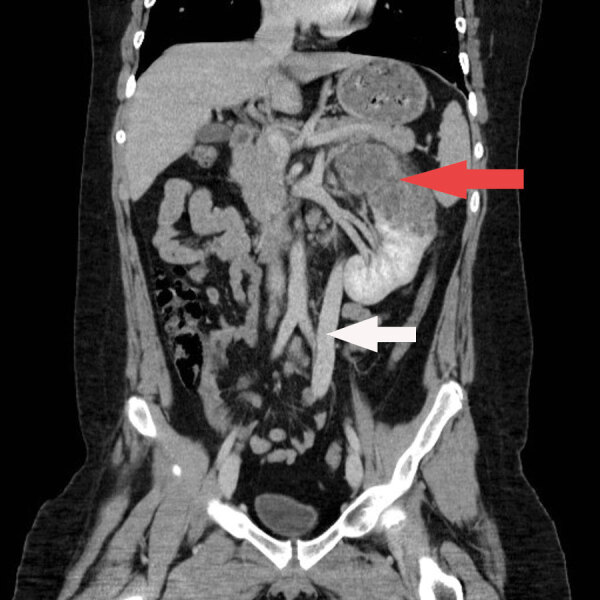
Coronal contrast enhanced CT (computed tomography) of the abdomen of a child with disseminated *Echinococcus multilocularis* infection without liver involvement, Canada, 2018. There is a large irregular hypodense left renal lesion (red arrow). A large porto-systemic shunt is partially visualized (white arrow).

Laboratory studies were notable for an erythrocyte sedimentation rate of 55 mm/h (reference <10 mm/h) and detection of erythrocytes and leukocyte esterase in the urine. Serology for HIV was negative, and lymphocyte subset values were within reference ranges. An ultrasound showed a large irregular echogenic lesion within the left kidney measuring ≈10 cm. A computed tomography (CT) scan of the abdomen showed an irregular cystoid mass measuring 8.2 × 8.7 × 12.3 cm, occupying the upper and interpolar regions of the left kidney. Magnetic resonance imaging (MRI) of the abdomen showed a heterogenous centrally necrotic renal mass exhibiting no notable central enhancement. Mild peripheral enhancement of the renal lesion was seen on MRI, likely corresponding to fibroinflammatory components (Figure 1).

CT of the chest and brain demonstrated numerous bilateral pulmonary nodules measuring <1.2 cm and a few small clustered ring-enhancing lesions in the left frontal lobe measuring <8 mm. These lesions were further characterized on brain MRI and again demonstrated ring enhancement, along with associated vasogenic edema (Appendix Figure). The patient underwent a biopsy of the renal lesion. CT of the abdomen also demonstrated a rare vascular anomaly of his portal system. The portal veins were diminutive in caliber, and a large caliber portosystemic shunt arose from the superior mesenteric vein, which drained into the right common iliac vein. This anomaly is consistent with a type II congenital portosystemic shunt (Abernethy malformation) (*4*).

The working diagnosis was tuberculosis. Samples from sputum, feces, urine, bronchoalveolar lavage, and renal biopsy did not show the presence of acid-fast bacilli on staining. Results of *Mycobacterium tuberculosis* PCR performed on the renal tissue and bronchoalveolar lavage sample were negative. The patient was initiated on antimycobacterial therapy while awaiting results from cultures, which were negative after 6 weeks of incubation. 

**Figure 2:**
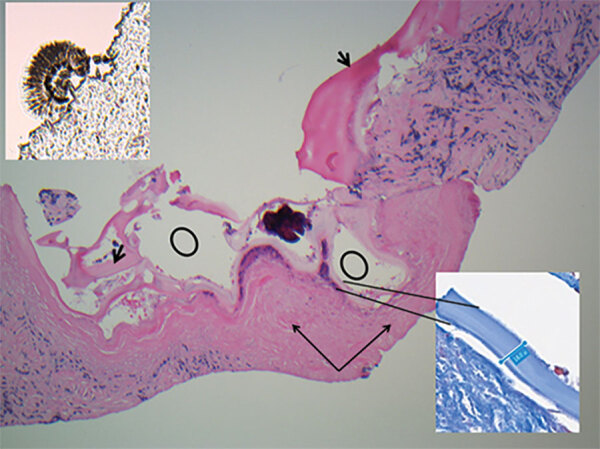
Kidney core biopsy of a child with disseminated *Echinococcus multilocularis* infection without liver involvement, Canada, 2018. Shown are folded laminated membrane (short black arrows) encircling variable-sized cystic structures (black circles) containing calcified and necrotic debris and dense periparasitic fibrosis (long black arrows), in a background of chronic inflammation and fibrosis. No residual normal kidney parenchyma was seen (hematoxylin and eosin stain, original magnification × 40). Inset at lower right shows laminar membrane, 18–19.4 μm in thickness in a background of fibrosis (Masson trichrome, original magnification ×40). Insert at upper left shows scolex attached to the paraffin edge of the block (original magnification ×40).

Under ultrasound, sections of core biopsies showed a predominantly solid lesion composed of dense fibroconnective tissue and containing macrophages, myofibroblasts, occasional multinucleated giant cells, and epithelioid histiocytes (Figure 2). In a few cores, there was evidence of variable-sized cystic structures encircled by a thin laminated layer with a thickness of 17–39.6 μm, as measured on Masson trichrome special stain (Figure 2). The luminal side of the cysts contained abundant dystrophic calcifications. A scolex was identified, which is uncommon (Figure 2) (*5*) and suggestive of a multicystic parasitic mass with associated periparasitic granulomatous inflammation, most supportive of *E. multilocularis* species. Echinococcal species serology using *E. granulosus* cyst soluble antigens was strongly positive.

We sent tissue samples to the National Reference Centre for Parasitology, where a PCR-based assay was positive for *E. multilocularis* infection (*6*). Treatment with albendazole was initiated, and tuberculosis medications were discontinued. We decided to proceed with medical therapy and defer any surgical intervention, given the exceedingly difficult surgical approach to perform a total nephrectomy while avoiding rupture of cyst contents. The BMI and the shunt also made surgery challenging, as did the location and numbers of other lesions.

Two years later, the patient is asymptomatic, and tolerating albendazole. Regular MRI imaging has shown no notable changes in the extent of the lesions.

## Conclusions

This case of disseminated AE has notable aspects. Cases of human AE are rare in Canada and in children. This case-patient had multisystem disease without evidence of hepatic involvement in the setting of a rare congenital portosystemic shunt.

The epidemiology of *E. multilocularis* parasites in Canada has been rapidly evolving over the past decade. Recent reports have identified additional areas of endemicity in wildlife, as well as human cases. Historically, affected wildlife were predominantly from the Arctic and far north or in the northern regions of central Canada, including Manitoba, Saskatchewan, and Alberta (*3**, **7**–**9*). Identification in wildlife from periurban environments is increasing. *E. multilocularis* parasites were detected in 23 of 91 canid carcasses collected within the Calgary and Edmonton metropolitan areas during 2009–2011 (*10*). Positive isolates from coyotes and deer mice in an urban region in Saskatchewan, as well as positive isolates from periurban areas of northern British Columbia, demonstrated a haplotype that was similar to some isolates from Europe (*7*,*11*). In an older study, 23% of coyotes tested in a rural national park in Manitoba were positive for *E. multilocularis* parasites (*9*). Recently, scat samples from 10 of 169 domestic dogs and wild canids from suburban parks around Winnipeg were positive (*12*). After a small cluster of domestic dogs that had diagnoses of *E. multilocularis* infection in Ontario, a survey of wild canid carcasses was conducted across southern Ontario. Of 460 fecal samples, 23% were positive by PCR for *E. multilocularis* DNA (*13*). Of the 18 affected public health units, 10 had clustering with higher prevalence and were in regions with human population densities of up to 1,700 persons/km^2^. Risk for human transmission clearly exists around several large urban centers in Canada.

Human AE, especially pediatric AE, has rarely been reported in Canada. Using hospital discharge data, we found no cases of AE documented in Manitoba during 2002–2012 and only 16 reported nationally (*14*). Alarmingly, during 2013–2020, a total of 7 human cases have been confirmed through the Alberta Public Health Laboratory, with 6 confirmed after 2016 (*15*). All were in adults, and all had hepatic lesions. In contrast, our patient was a child with no evidence of liver involvement despite extensive extrahepatic disease. In previous large case series, both aspects were unusual (*16*). A European registry of 559 patients had only 4 reported as children (*17*). Only 13 of the 559 patients lacked any liver involvement.

Factors favoring liver involvement are poorly understood. Our case illustrates an additional mechanism for extrahepatic AE involvement. We suggest that having a congenitally hypoplastic or absent portal vein can be a contributor for extrahepatic AE. In our case, the oncosphere released by ova invaded the small intestine and traveled through the superior mesenteric veins and into the large portosystemic shunt by path of least resistance. The portosystemic shunt drains directly into the right common iliac vein, providing a route for spread to different organs, including the kidneys, lungs, and brain in our case.

In summary, we describe a challenging case of AE and emphasize the difficulty in establishing the diagnosis given the lack of liver involvement, age of the patient, and context of local epidemiology. When viewed in the scope of the recent literature, the epidemiology of AE in Canada is evolving, including our understanding of trends involving suburban regions with large human populations. Surveillance using a One Health approach would help in planning for the future.

Appendix Brain MRI of a child with disseminated *Echinococcus multilocularis* infection, Canada, 2018.
