# Supplementary material for: Disseminated Echinococcus multilocularis Infection without Liver Involvement in Child, Canada, 2018
Source: Emerg Infect Dis. 2020 Aug;26(8):1856–9. doi: 10.3201/eid2608.191644 (PMC7392456; doi:10.3201/eid2608.191644)
Supplement: Appendix — Brain MRI of a child with disseminated Echinococcus multilocularis infection, Canada, 2018. [file 19-1644-Techapp-s1.pdf]

Article DOI: https://doi.org/10.3201/eid2608.191644

Disseminated *Echinococcus multilocularis* Infection without Liver Involvement in Child, Canada, 2018

Appendix

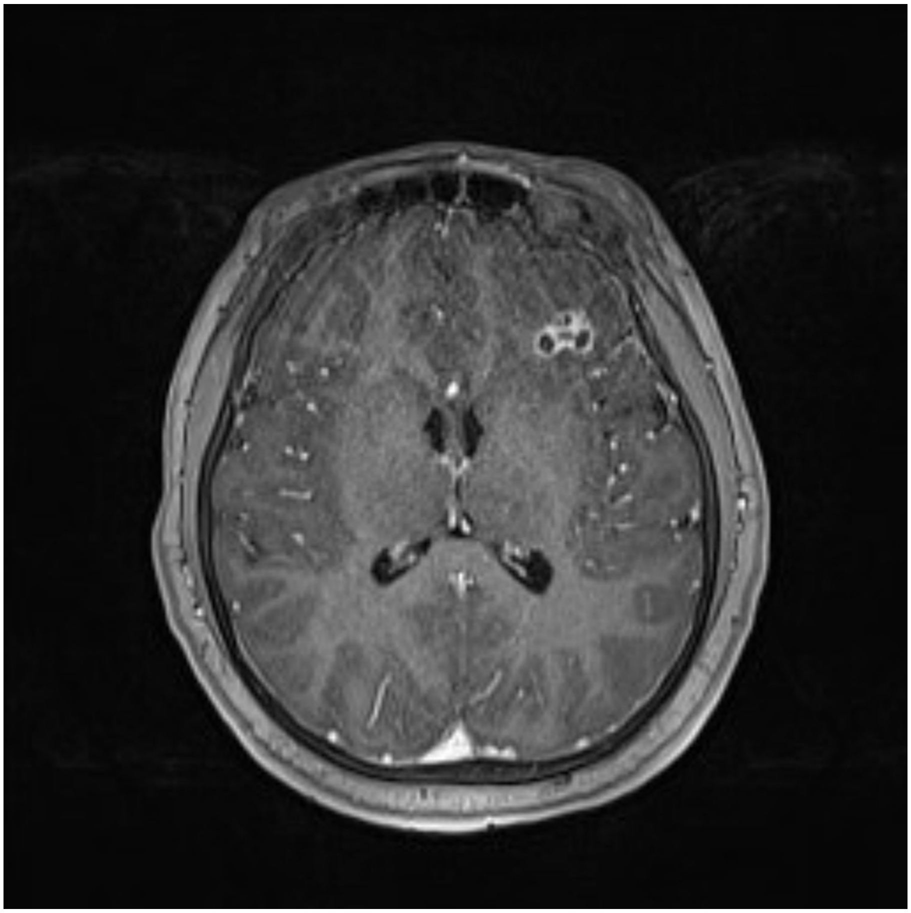

Appendix Figure. Brain MRI of child with disseminated *Echinococcus multilocularis* infection, axial T1 postcontrast image. There is a cluster of small ring-enhancing cavities within the left frontal lobe, surrounded by vasogenic edema.
